# Leveraging the Multidisciplinary Tumor Board for Dissemination of Evidence-Based Recommendations on the Staging and Treatment of Gastric Cancer: A Pilot Study

**DOI:** 10.1245/s10434-022-12628-4

**Published:** 2022-10-12

**Authors:** Shivani N. Mehta, Edna C. Shenvi, Sarah L. Blair, Abigail Caudle, Lisa M. Lowenstein, Kaitlyn J. Kelly

**Affiliations:** 1 Department of Surgery, University of California, San Diego, CA USA; 2 Department of Surgery, University of Texas MD Anderson Cancer Center, Houston, TX USA

## Abstract

**Background:**

Compliance with evidence-based treatment guidelines for gastric cancer across the United States is poor. This pilot study aimed to create and evaluate a change package for disseminating information on the staging and treatment of gastric cancer during multidisciplinary tumor boards and for identifying barriers to implementation.

**Methods:**

The change package included a 10-min video, a brief knowledge assessment, and a discussion guide. Commission on Cancer-accredited sites that perform gastrectomy were invited to participate. Participants completed the Organizational Readiness for Implementing Change (ORIC) scale (range, 12–60) and scales to measure the feasibility, acceptability, and appropriateness (score range, 4–20). Semi-structured interviews were conducted to further define inner and outer setting barriers.

**Results:**

Seven centers participated in the study. A total of 74 participants completed the pre-video knowledge assessment, and 55 participants completed the post-video assessment. The recommendations found to be most controversial were separate staging laparoscopy and modified D2 lymphadenectomy. Sum scores were calculated for acceptability (mean, 17.43 ± 2.51) appropriateness (mean, 16.86 ± 3.24), and feasibility (mean, 16.14 ± 3.07) of the change package. The ORIC scores (mean, 46.57 ± 8.22) correlated with responses to the open-ended questions. The key barriers identified were patient volume, skills in the procedures, and attitudes and beliefs.

**Conclusions:**

The change package was moderately to highly feasible, appropriate, and acceptable. The activity identified specific recommendations for gastric cancer care that are considered controversial and local barriers to implementation. Future efforts could focus on building skills and knowledge as well as the more difficult issue of attitudes and beliefs.

Gastric cancer is the fifth most common cancer and the third leading cause of cancer death worldwide.^1^ Although its incidence is relatively low in the United States, the incidence of poorly differentiated, diffuse-type disease is increasing.^1^ Multiple clinical trials conducted in recent years have aimed to optimize multidisciplinary treatment (MDT) and outcomes for gastric cancer patients. Despite these studies and subsequent updates to evidence-based treatment guidelines, long-term oncologic outcomes for patients with all but the earliest stage of disease have not improved significantly in decades, particularly in the United States.^2^

The reasons for stagnant outcomes are likely multifactorial, but potentially include delay or outright failure to incorporate trial results and evidence-based guidelines into clinical practice. Studies have shown that compliance with evidence-based staging and treatment recommendations for gastric cancer, including both surgical and perioperative components of care, is low in the United States. Worhunsky et al.^3^ reported that in the early 2000’s, only 45% of patients with gastric cancer in California received stage-specific therapy in accordance with the National Comprehensive Cancer Network (NCCN) treatment guidelines. A more recent study by Zhao et al.^4^ using the National Cancer Database (NCDB) showed that only about 40% of patients with potentially curable disease treated from 2004 to 2014 met surgical standards, even while receiving treatment at Commission on Cancer (CoC)-accredited centers.

Surgical standards for gastric cancer include performance of a staging laparoscopy (SL) with peritoneal lavage to evaluate for radiographically occult peritoneal disease and confirm its clinical stage.^2,5^ This is recommended to be performed at baseline, before a treatment plan is formed, for clinical stage 2 or 3 patients for whom curative-intent therapy is planned. Additional surgical standards include modified D2 lymphadenectomy (with removal of at least 16 lymph nodes), total gastrectomy for proximal cancers, and distal/subtotal gastrectomy for distal cancers.^5^ Findings have shown that compliance with these surgical standards is associated with improved survival.^4^ In addition to surgical standards, perioperative systemic therapy is recommended for patients with clinical T3/4 or node-positive disease and has been confirmed to improve survival outcomes in randomized controlled trials.^6,7^ If only approximately 40% of gastric cancer patients are receiving guideline-concordant care in the United States, there is a need to understand and address the barriers to incorporation of treatment recommendations at the local level.

A behavioral change intervention is a coordinated set of activities designed to change specified behavior patterns.^8^ Multidisciplinary tumor boards are an established quality activity in cancer care, and are associated with increased compliance with national guidelines, changes in treatment plans in 40% of presented cases, and potentially improved oncologic outcomes for patients.^9–14^ In addition, tumor boards are part of existing infrastructure at CoC-accredited centers, presenting an opportunity where multiple stakeholders are present in a single setting.

This pilot study aimed to create and evaluate a change package to disseminate information on the staging and treatment of gastric cancer during multidisciplinary tumor board meetings. An additional aim was to develop tools to guide discussion around organizational practice changes that account for the contextual framework of communities with heterogeneous resources.

## Methods

The protocol for this study was submitted for review to the University of California San Diego Institutional Review Board (IRB), was determined not to be human subjects research, and therefore was exempt from ongoing review.

### Change Package

The change package consisted of a 10-min video summarizing current evidence-based recommendations for the staging and treatment of gastric cancer. The video can be accessed at

https://vimeo.com/343496200/763c6fdf45. The video also contained a brief quiz with five questions for knowledge assessment given before and after the video, and a discussion guide to facilitate group discussion at the tumor board meeting.

All the individual participants were asked to complete pre- and post-video quizzes, and a single designated member from each tumor board was asked to complete the accompanying discussion guide. At the pre-video quiz, the participants also were asked whether they would be willing to participate in a brief semi-structured phone interview after the activity.

### Participating Sites

To be eligible, sites had to be CoC-accredited and perform gastrectomy for cancer. The study was initially planned to include centers in Southern California within a 100-mile radius of the University of California San Diego, with the package to be delivered via an in-person site visit. Cancer liaison physicians and registrars at 15 potential sites were contacted by e-mail first in October 2020 to explain the study and request participation. Because of the COVID-19 pandemic, many sites responded that tumor boards were on hold or being restructured. The change package was then similarly restructured to be made available virtually.

Sites were contacted again in January 2021, and that time as an incentive, it was explained that participation in the study might satisfy CoC standards 7.4 or 8.1. Because of low accrual, it was decided to expand the solicitation e-mail to state chairs in a broader region of the Southwest (24 additional sites were contacted). Finally, the study was advertised by the American College of Surgeons Cancer Research Program (ACS CRP) in a Cancer News e-mail to all CoC-accredited sites in the United States.

Once a site confirmed participation and established a date for their tumor board meeting, the pre-video knowledge assessment was administered to the site participants via Google Docs. After these were completed, the video link and post-video knowledge assessment were distributed. Sites were given the freedom to choose whether to allow participants to watch the video and complete the post-video questions individually or to show the video at the tumor board meeting. The discussion guide also was sent via Google Docs to facilitate a group discussion at the tumor board.

### Study Design and Statistics

The primary end point of the study was the feasibility, acceptability, and appropriateness of the change package. These were assessed by the Feasibility of Intervention (FIM), Acceptability of Intervention (AIM), and Intervention Appropriateness (IAM) measures.^15^ Although cut-off scores for interpretation have not been established to date, higher scores on the scale (from 4 to 20) indicate higher feasibility, acceptability, and appropriateness.

The Organizational Readiness for Implementing Change (ORIC) Scale was administered as part of the discussion guide and used to assess change readiness at participating sites.^16^ This scale is composed of two subscales: change commitment (range, 6–30) and change efficacy (range, 6–30). The total summed possible score range is 12 to 60, with higher scores indicating greater readiness for change.

Descriptive statistics were used to compare these measures as well as baseline characteristics of the sites. Semi-structured interviews were conducted based on the five domains of the Consolidated Framework for Implementation Research (CFIR).^16,17^ All interviews were conducted by a single investigator (K.K.). The interviews were recorded with Re-CallRecorder software and transcribed for subsequent analysis. Verbal consent was obtained from all subjects before recording. A mixed-methods analysis was performed using the AIM and ORIC results as well as single coding of semi-structured interviews for themes.

## Results

### Participants

Seven centers participated in the study: one academic, one comprehensive community, two community, and three hospital associate centers. The characteristics of the centers are summarized in Table 1.

**Table 1:** Characteristics of seven participating sites

Site	CoC cancer program type	Gastric cancer patients per year	Activity at tumor board	Pre-quiz participants	Post-quiz participants
1	Community	20*–*30	No	15	1
2	Academic comprehensive	>30	No	1	5
3	Hospital associate	<10	Yes	14	11
4	Comprehensive community	<10	Yes	7	7
5	Hospital associate	10*–*20	Yes	19	19
6	Hospital associate	10*–*20	Yes	3	2
7	Community	>30	Yes	15	10

*CoC* Commission on cancer

The centers reported seeing anywhere from fewer than 10 to more than 30 gastric cancer patients per year. Gastrectomies were performed at all the centers. Compliance with the knowledge assessment quiz was better before the video than afterward.

**Fig. 1:**
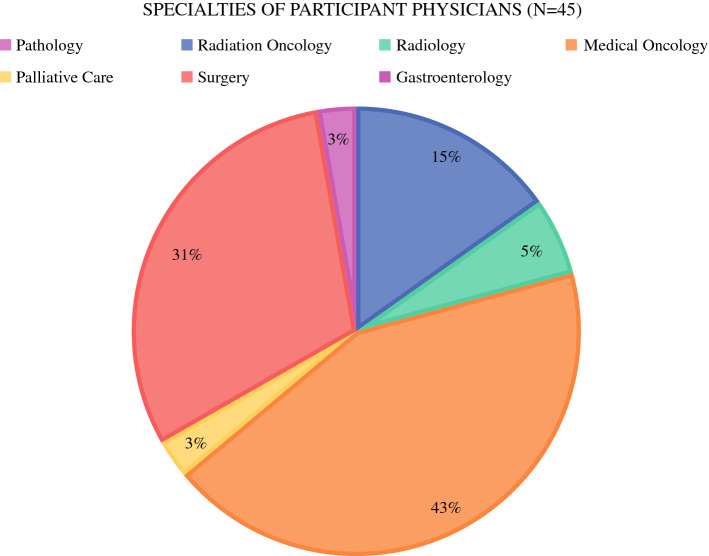
Pie graph illustrating the medical specialties of the physicians who participated in the tumor board activity among the seven sites

Of the 74 individual participants, 45 (61%) were physicians (MD degree), 3 (4%) were advance practice providers (PAs or NPs), 10 (14%) were nurses (RNs), 3 (4%) were genetic counselors, and the remaining 13 (21%) were research, administrative, and supportive staff. The areas of specialty of the MD participants are shown in Fig. 1. The majority were medical oncologists (31%), pathologists (27%), or surgeons (22%).

### Pre- and Post-Video Quiz

**Fig. 2:**
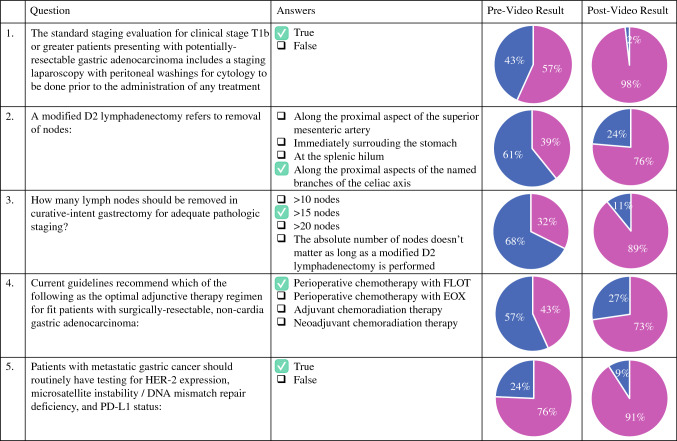
Table showing the five knowledge assessment questions together with the pre- and post-video results for each question

The questions included in the quiz and the responses before and after the video are summarized in Fig. 2. Whereas 74 participants completed the quiz before watching the video, only 55 completed it again afterward. The percentage of correct responses improved for all questions after the video, but no question was answered correctly by 100% of participants, even after the video.

For question 1 about conducting an SL with peritoneal washings for cytology before treatment, 57% of the participants answered correctly before the video versus 98% afterward. For question 2 regarding the definition of D2 lymphadenectomy, 39% of the participants answered correctly before the video versus 76% afterward. For question 3 regarding the number of lymph nodes recommended for appropriate staging, 32% answered correctly before the video versus 89% afterward. For question 4 regarding the current guidelines for the optimal adjunctive therapy regimen for resectable gastric adenocarcinoma, 43% answered correctly before the video versus 73% afterward. For question 5 regarding indications of a routine test for epidermal growth factor receptor 2 (HER2) expression, microsatellite instability/DNA mismatch repair deficiency, and PD-L1 status, 76% of the participants answered correctly before the video versus 91% afterward.

### Discussion Guide Responses

The primary end point of the study was the feasibility, acceptability, and appropriateness of the change package. These were measured by a validated scale with possible scores of 0 to 20.^15^ The change package was moderately to highly acceptable (mean, 17.43 ± 2.51), feasible (mean, 16.14 ± 3.07), and appropriate (mean, 16.86 ± 3.24). The recommendations discussed in the video that were identified as the most controversial by the participating sites were those for a separate staging laparoscopy with washings (3 sites) and for modified D2 lymphadenectomy (3 sites). The participating academic center reported that they found none of the recommendations to be controversial.

The ORIC instrument change commitment and change efficacy scores were calculated for each site and are shown in Table 2 together with the individual instrument questions. A total sum score also was calculated. The overall mean score for change commitment among all the sites was 23.72 ± 4.53 and for change efficacy was 22.85 ± 4.02, with a net mean score of 46.57 ± 8.22. The scores for the individual sites together with site-reported barriers to incorporation of recommendations are summarized in Table 3. The most commonly reported barriers to implementation of the recommendations in the video were low patient volume, lack of practitioners skilled in procedures, strongly held beliefs, and concerns about validity of the guidelines.

**Table 2:** Organizational readiness for change responses at seven participating sites

ORIC instrument statements	Disagree	Somewhat disagree	Neither agree nor disagree	Somewhat agree	Agree
*Change commitment*					
People who work here feel confident that the organization can get people invested in implementing this change	0	0	4	0	3
People who work here are committed to implementing this change	0	0	2	3	2
People who work here feel confident that they can keep track of progress in implementing this change	0	0	3	2	2
People who work here will do whatever it takes to implement this change	0	0	2	2	3
People who work here feel confident that the organization can support people as they adjust to this change	0	0	1	4	2
People who work here want to implement this change	0	0	2	1	3
*Change efficacy*					
People who work here feel confident that they can keep the momentum going in implementing this change	0	0	2	4	1
People who work here fee confident that they can handle the challenges that arise in implementing this change	0	0	3	3	1
People who work here are determined to implement this change	0	0	3	3	1
People who work here feel confident that they can coordinate tasks so that the implementation goes smoothly	0	0	2	4	1
People who work here are motivated to implement this change	0	0	3	3	2
People who work here feel confident that they can manage the politics of implementing this change	0	0	3	3	2

**Table 3:** Summary of ORIC scale results and self-reported barriers at individual sites

Site	Change commitment	Change efficacy	Total	Identified barriers to implementation of recommendations
1	23	21	44	Availability of surgeons skilled in D2 dissection Too few gastric cancer cases per year for a dedicated MDT
2	30	30	60	Patients referred too late in the course
3	20	19	39	Availability of surgeons skilled in D2 dissection
4	21	24	45	Too few gastric cancer cases per year to have a dedicated MDT
5	25	24	49	Strongly held beliefs by colleagues/partners Concerns about validity of current guideline recommendations or disagreement with them
6	29	24	53	Strongly held beliefs by colleagues/partners
7	18	18	36	Too few gastric cancer cases for dedicated MDT

*ORIC* Organizational readiness for implementing change; *MDT* Multidisciplinary team

### Semi-Structured Interviews

Semi-structured interviews were conducted with five individuals interviewed from four of the participating sites. The key quotations from these interviews pertinent to the intervention characteristics as well as outer and inner setting factors are summarized in Table 4. The common themes from these interviews were regarding the change package itself, including use of the influence of a multidisciplinary setting on decision-making and communication and the impact of concise delivery of pertinent information.

**Table 4:** Selected quotations from semi-structured interviews conducted according to the CFIR domains

CFIR domain	Qualitative data analysis	
Intervention characteristics	Theme	Influence of multidisciplinary tumor board on decision-making and communication
	Selected quotations	“I think that presenting it in a vacuum to either just surgeons or just medical oncologists etc. would be a bigger challenge but when presented at a TB or multidisciplinary meeting, I think you get more robust discussion that will lead to different pieces of the group to identify areas of improvement.” (subject #3)
		“I think it’s important that it was presented in a multidisciplinary forum . . . about lymph node numbers and totals, some of the surgeons said ‘well, I always get that many’, but then the pathologists were like ‘well, I don’t know that I always see that many nodes’, and then they were talking about how they gross specimens and how they identify the lymph nodes and stuff and I think it was a very robust conversation that way” (Subject #1)
	Theme	Impact of concise delivery of pertinent information
	Selected quotations	“I thought that the speakers were intelligent and competent but they weren’t the type that throw information and numbers at people and are intimidating. You could tell that they were very comfortable with the subject matter and presented it in a way that was very acceptable. . . . There were several comments made like ‘I didn’t know we should be doing that’, ‘wow were you aware of that’? (subject #3)
		“But to hear some of the general surgeons in the room realize that ‘oh wow, maybe we haven’t been doing things right’. I thought those were pretty important statements to make just after having watched a short video.” (subject #4)
		In private practice, people are really busy. They are dealing with more than just the clinical stuff. They’re dealing with maintaining their relationships. If you think that politics in academics are something, you should see it in the community. You know community surgeons just spend a lot more time just tracking down patient data. That’s time that I think in academia, people can be using to just read and take in information. So you know, that said, I think if you can give people stuff that’s relevant and immediately pertinent to a problem that they’re having, then it’s very impactful and well received. (subject #1)
Outer setting	Theme	Thoughts about referring complex cancer patients to higher-volume centers
	Selected quotations	“There were some surgeons who said, ‘you know I wouldn’t send these complex cancer cases out because that’s a big revenue loser’. . . . They worry about their referral base because they’ve had other docs in the community sending them livers and gastric cancer for years and now all of the sudden they’re saying ‘we’re sending this to a tertiary center’. You know, there’s a certain look to that in private practice.” (subject #5)
		“In our group, many of our surgeons are basically eat what you kill so it’s hard for them to send to tertiary centers.” (Subject #2)
	Theme	Concerns about strength of the data
	Selected quotations	“Someone said I don’t think staging laparoscopy is being done routinely like that at a lot of other places. And someone else looked this up during the conversation and pointed out that it is only a category 2B recommendation by the NCCN.” (subject #2)
		“Several in our group brought up that some of this is still controversial and hasn’t been entirely proven. Like doing the laparoscopy separately and the extent of lymph node dissection.” (subject #5)
Inner setting	Theme	Influence of individual provider practices and skill levels
	Selected quotations	“I think other barriers this may encounter is certain level of expertise. Now the extent of lymph node dissection, you know, some said gosh, you know, I haven’t done that in years, that’s a lot of surgery, I don’t know if I’d be willing to go that far. And that’s when someone suggested, you know, ‘what if we send those out’. And then you get into what a hospital system is capable of. You know taking care of, from a nursing and facility standpoint.” (subject #4)
		“High-quality D2 dissection is difficult to achieve when not all surgeons know the technique, logic, gastric lymphatic anatomy, history, development and international data of D2 dissection. This is a lot to keep up with when we don’t see many patients with gastric cancer.” (subject #3)

*CFIR* Consolidated framework for implementation research; *TB* Tumor Board; *NCCN* National comprehensive cancer network

Regarding too few patients for a dedicated team, one interviewee expanded on what it is like to be in private practice and how there is very little time for reading and keeping up with the literature. Others stated that after watching the video, clinicians have responses such as “oh wow, maybe we haven’t been doing that right. I thought those were pretty important statements after just having watched a short video.”

Another inner setting theme was the influence of individual provider practices and skill level on care. One interviewee stated, “High quality D2 dissection is difficult to achieve when not all surgeons know the technique, logic, gastric lymphatic anatomy, history, development, and international data of D2 dissection. This is a lot to keep up with when we don’t see many patients with gastric cancer.” Still another stated, “Now the extent of lymph node dissection, you know, some said gosh you know I haven’t done that in years, that’s a lot of surgery, I don’t know if I’d be willing to go that far.”

Outer setting themes included thoughts about referring complex cancer patients to high-volume centers. The interviews shed light on thoughts about this with quotations such as, “in our group, many of our surgeons are basically eat what you kill so it’s hard for them to send to tertiary centers,” and “they worry about their referral base because they’ve had other docs in the community sending them livers and gastric cancer for years and now all of the sudden they’re saying we’re sending this to a tertiary center. You know, there’s a certain look to that in private practice.”

An additional outer setting theme focused on concerns about the validity of the data. One interview participant stated, “Several in our group brought up that some of this is still controversial and hasn’t been entirely proven. Like doing the laparoscopy separately and the extent of lymph node dissection.”

## Discussion

This change package aimed at disseminating current staging and treatment information on gastric cancer and generating discussion in a multidisciplinary setting was uniformly well received at the participating centers. The quantitative feedback showed that it was moderately to highly acceptable, feasible, and appropriate. These measures were the primary outcome of the study and are accepted as leading indicators of implementation success.

The ORIC instrument, used for an additional study end point, measures “the extent to which organizational members are psychologically and behaviorally prepared to implement organizational change.”^18^ On this scale of 12 to 60, the scores at the participating centers ranged from 36 to 60, indicating a moderate-to-high level of readiness to implement change. Also of note, no participants selected a response of “disagree” or “somewhat disagree” to any of the ORIC instrument questions (Table 2).

The recommendations from the video reported to be the most controversial were those for baseline SL with peritoneal lavage and modified D2 lymphadenectomy. This was not surprising because these have been controversial topics for decades. Radiographically occult peritoneal metastatic disease is present in more than 30% of patients considered for curative-intent treatment of gastric cancer.^19^ The peritoneum is the most common site of metastasis, with recurrence and microscopically positive peritoneal washings constituting stage 4 disease. Baseline SL with washings is necessary for complete staging information for all patients with T1b or greater disease or node-positive disease.^2,20^ Despite this fact, clinicians have long resisted performing baseline SL, both in day-to-day practice and in clinical trials.^4^ The SWOG S0425 study of neoadjuvant chemoradiation for patients with surgically resectable gastric cancer was opened in 2006 and ultimately closed due to poor accrual in 2008 after reaching only 9% of the target accrual (NCT00335959). The reason for the poor accrual was thought to be the requirement of SL in the study protocol. Since that time, none of the completed large clinical trials analyzing neoadjuvant or perioperative therapy regimens around curative-intent gastrectomy have required SL, but finally, the ongoing CRITICS II trial does mandate it (NCT 02931890).^6,7,21,22^

In terms of node dissection, modified D2 lymphadenectomy, preserving the spleen and pancreatic tail, is the established standard of care in curative-intent gastrectomy and improves disease specific survival.^23,24^ It is easier and lower risk, however, to perform a gastrectomy without clearing these nodes. Surgeons trained to perform gastrectomy for benign disease or non-adenocarcinoma indications may not be comfortable with this more extended nodal dissection or even aware of the steps it entails.

Notably, both SL and modified D2 dissection are surgical standards. Recommendations on perioperative chemotherapy were not reported to be controversial by any of the participating sites. This could have been because perioperative therapy regimens are supported by relatively recent randomized prospective trials.^6^ Surgical standards often are not supported by such high-level evidence, but more often come from abundant retrospective data and consensus agreement.^5,25^ Concerns about validity of evidence or disagreement with it and strongly held beliefs by colleagues and partners were among the barriers to implementation reported by several sites.

Although the impact of SL on survival has not been demonstrated in the form of a prospective trial, SL it is supported by abundant literature and is necessary for guiding appropriate treatment.^19,26–28^ When SL is not performed at baseline and patients undergo systemic therapy, peritoneal disease present initially may respond and no longer be detectable, but the patient still will be at a much higher risk of recurrence because they were still stage 4 at baseline. These patients should go on to be considered for alternative therapies or clinical trials (e.g., targeted regimens or hyperthermic intraperitoneal chemotherapy [HIPEC]) or at a very minimum should have an informed discussion about their higher risk of recurrence and their prognosis before undergoing gastrectomy. Confirming a patient’s disease stage before initiation of invasive treatment with stated curative intent is a basic tenet of cancer care. In a randomized trial, D2 lymphadenectomy was examined, but it was decades ago, and the current recommendation for pancreas and spleen-sparing modified dissection are based on interpretation of those results, which may be why some still find this recommendation controversial.^24,29^

Additional barriers reported by multiple sites in this study were “the availability of surgeons skilled in D2 dissection” and “having too few gastric cancer cases per year for a dedicated team.” These topics came up in the semi-structured interviews and led to discussions about possibly referring gastric cancer patients to higher-volume centers. These quotations and the themes pulled from the interviews highlight issues in real-world practice that may be affecting care and outcomes for patients, particularly those with relatively rare cancers.

## Study Limitations

The greatest limitation of this study was the low number of participating sites. The study was advertised to all CoC-accredited centers that perform gastrectomy across the United States. The incentive provided was that participation could potentially fulfill one of two CoC accreditation metrics, but only seven sites participated. In the model of behavior described by Michie et al.,^8^ capability, opportunity, and motivation are at the center of what generates behavior (COM-B). It seems most likely that lack of motivation was the cause of the low accrual because all potential sites were capable of participating and were provided the opportunity to participate. Some prospective sites did report that they had insufficient time to spare in their tumor board meetings, so capability also may have been a factor.

Of the seven participating sites, six were non-academic sites and one was academic. It is noteworthy that the academic site reported the highest possible scores on both the acceptability/feasibility/appropriateness measures and the ORIC instrument, and that “patients referred too late in the course” was the only barrier to implementation. The non-academic sites all reported internal barriers, as described earlier. With so few participants, however, it was not possible to know whether this type of finding really is a trend or just a coincidence.

An additional limitation was the post-video quizzes. Many sites reported that the post-video quiz was not reliably accessible because all the participants were trying to take it simultaneously, limiting participation and completeness of the post-video quiz.

## Summary

In this novel pilot study, a change package on the staging and treatment of gastric cancer was created and evaluated. At its inception, the change package was intended to be delivered in person as an educational outreach visit, which is a validated methodology for affecting practice change.^30^ Because of the COVID-19 pandemic, the change package was converted to a virtual format that allowed broader delivery and inclusion. The change package was moderately to highly feasible, acceptable, and appropriate at the participating sites, and all the sites showed moderate to high readiness for implementing change. A major strength of the activity was the use of the multidisciplinary tumor board as the setting. The change package identified important barriers to implementation and generated robust discussion about these barriers and how they could possibly be overcome.

## Conclusions

This change package design is well suited for dissemination of information on the staging and treatment of a relatively rare cancer, particularly to low-volume centers in the United States. Further studies like this investigation are needed, ideally on a larger scale, to validate these pilot data for understanding what types of factors influence patient care at different hospital types, and to identify targets for improving compliance. It may be necessary to provide further incentive to participating sites. Studies have shown that compliance with cancer surgery standards is associated with improved oncologic outcomes for many cancer types, including colon, breast, and stomach cancers.^4,31,32^ As more surgical standards are developed and converted into CoC metrics, it is increasingly important to understand how to improve compliance so that cancer patients can benefit from these efforts and receive high-quality care.^16^
